# First Trimester Echogenic Lung Lesions: A Diagnostic Challenge and Review of Differential Diagnoses

**DOI:** 10.7759/cureus.92114

**Published:** 2025-09-12

**Authors:** Chameli Subbaraj, Mustafa Guma

**Affiliations:** 1 Obstetrics and Gynaecology, Basildon University Hospital, Basildon, GBR; 2 Fetal Medicine, Basildon University Hospital, Basildon, GBR

**Keywords:** congenital high airway obstruction syndrome, cystic adenomatoid malformation, echogenic fetal lungs, pulmonary sequestration, turner’s syndrome

## Abstract

Foetal echogenic lungs are characterised by the appearance of small bright spots within the foetal lungs observed via antenatal ultrasound, which may indicate underlying developmental abnormalities. The presence of echogenic lungs is a rare sonographic finding identified during the first trimester of pregnancy. In this report, we present the case of a healthy 25-year-old primigravida who was found to have multiple foetal abnormalities in her routine first-trimester scan, following which she was scanned by the foetal medicine team, who confirmed the finding of foetal echogenic lungs along with cystic hygroma and hydrops foetalis. Turner's syndrome was confirmed with successive invasive tests, and the pregnancy was terminated at 14 weeks. Our findings not only contribute to the limited data on this rare sonographic occurrence but also highlight the importance of vigilant foetal monitoring in cases of echogenic lungs, allowing for timely intervention and management strategies. This case strongly supports the need for further research into the implications of echogenic lung findings and their association with chromosomal abnormalities.

## Introduction

Foetal echogenic lung lesions (ELL) represent a characteristic finding that can be identified as early as first-trimester ultrasound examinations. This finding is particularly associated with either airway obstruction or intrinsic lung lesions. Airway obstruction includes CHAOS (congenital high airway outlet obstruction syndrome), which is characterised by complete or near-complete obstruction of the foetal airway, and it can be subdivided into tracheal atresia, congenital tracheal stenosis, laryngeal atresia, and congenital laryngeal stenosis. Intrinsic lung lesions include congenital cystic adenomatoid malformation (CCAM: a condition where normal lung tissue is replaced by fluid-filled cysts) and pulmonary sequestration (PS: a birth defect where the lung tissue is nonfunctional). The reason why these occur is unclear. Until recently, it was thought to occur as an isolated lesion rather than as a part of the syndrome, but after a thorough review of the existing literature, it was evident that only one case of this condition associated with Turner's syndrome has been documented prior to our study [1]. In this report, we present the second case of a foetus with an ELL, detected antenatally by ultrasound at 13 weeks and two days of gestation in a 25-year-old primigravida. Subsequent invasive testing confirmed the diagnosis of Turner’s syndrome, leading the patient to make the difficult decision of terminating her pregnancy. This case enlightens the importance of early detection and the potential implications of echogenic lung findings.

## Case presentation

A 25-year-old primigravida of body mass index (BMI) 34.5 kg/m² with an A negative blood group had her routine first trimester scan at 12+6 weeks. The foetal heart rate was 178 bpm (normal 110-160 bpm), and the nuchal translucency was 14 mm (normal <3.5 mm), along with severe cystic hygroma and generalised oedema, and she was hence referred to the Foetal Medicine unit (FMU).

She had her first trimester screening on the same day as her scan. Free beta human chorionic gonadotropin (BHCG) and pregnancy-associated plasma protein-A (PAPP-A) were 1.2 multiples of median (MoM) and 0.55 MoM, respectively. The chance of Down’s, Edward’s, and Patau’s syndrome was >1 in 2.

**Figure 1:**
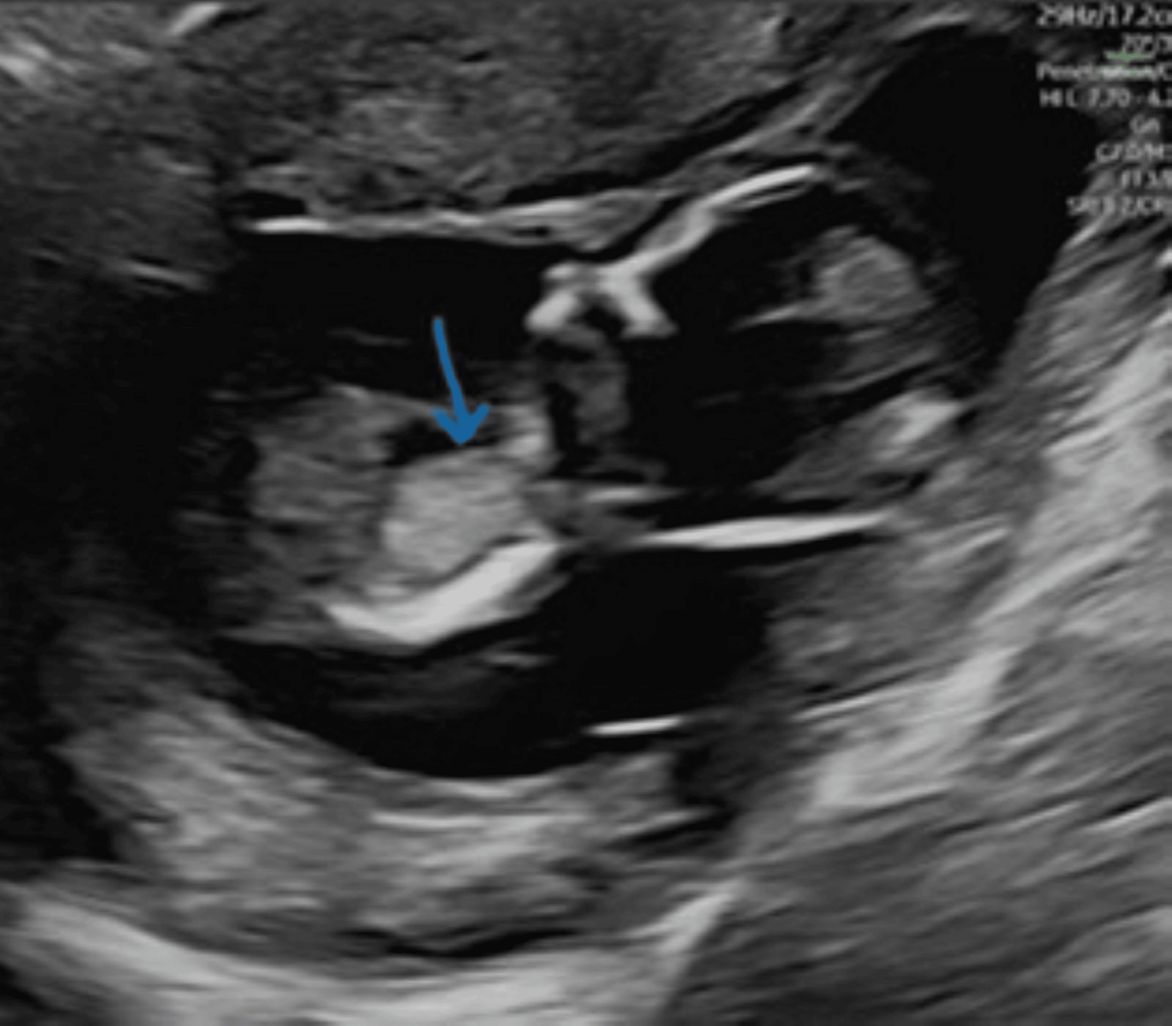
Turner's syndrome with echogenic lungs in the midsagittal plane (arrowhead)

**Figure 2:**
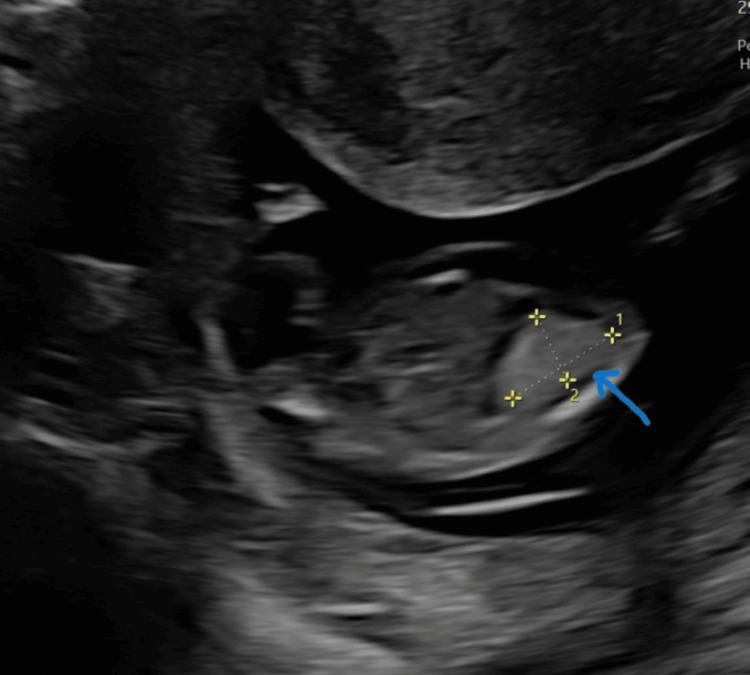
Echogenic lesion in lungs at 13+2 weeks in coronal plane (arrowhead)

**Figure 3:**
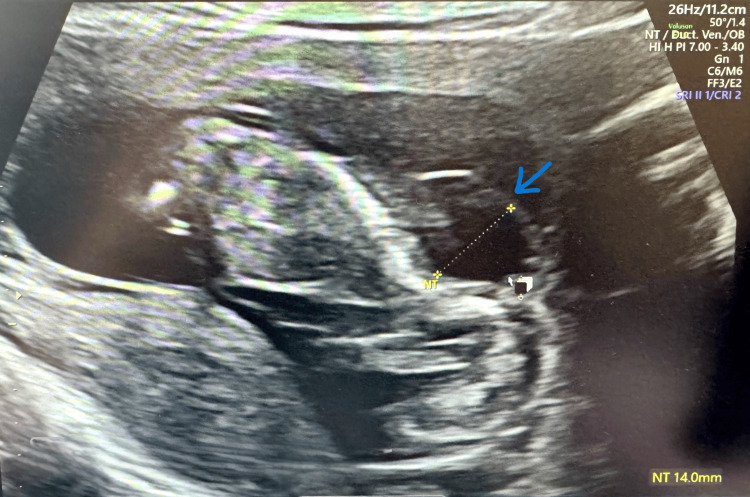
Image showing nuchal translucency with cystic hygroma (arrowhead)

**Figure 4:**
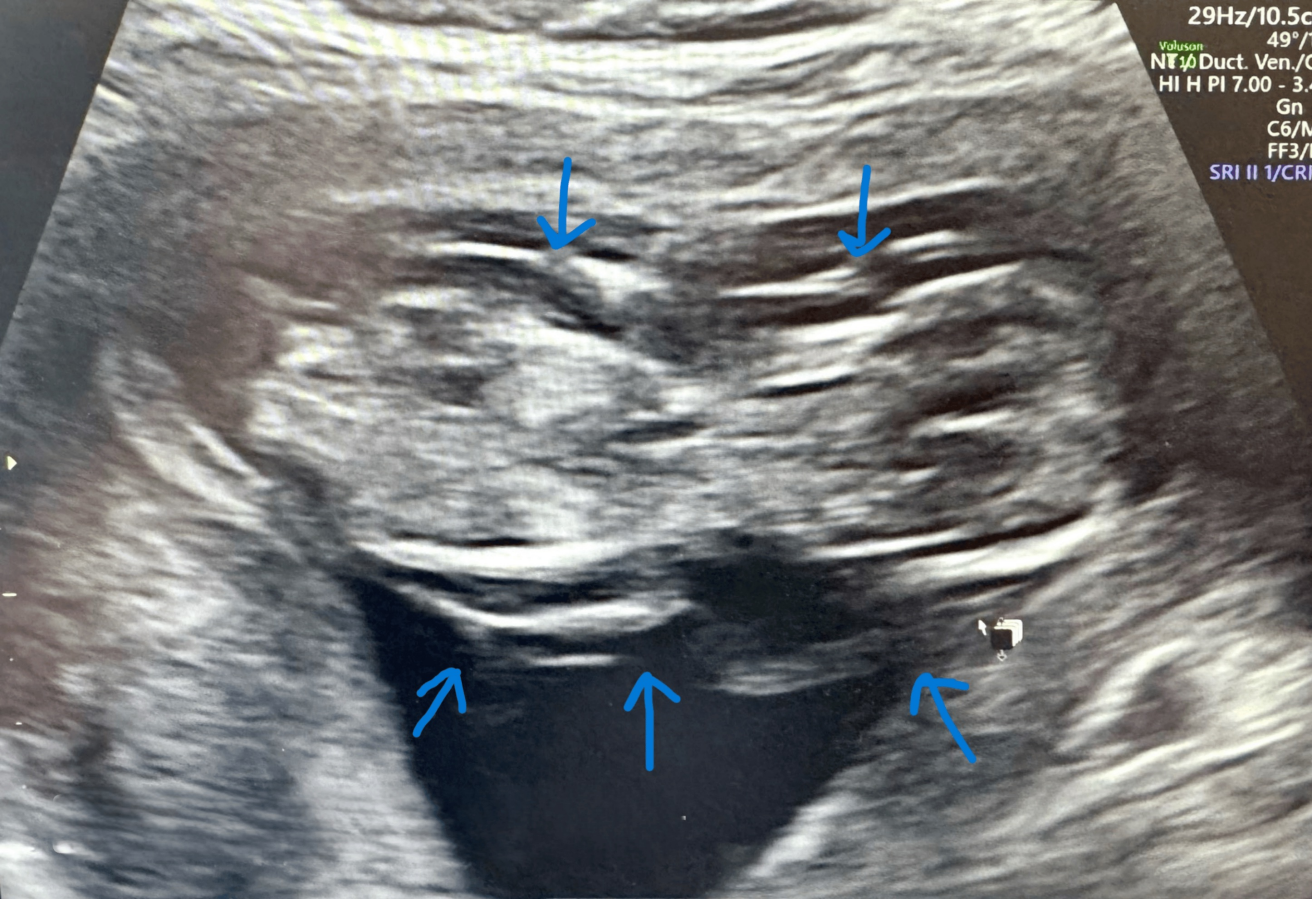
Image showing generalised oedema (arrowhead) – hydrops fetalis

Her next scan was at 13+2 weeks with the Foetal Medicine team. The scan identified an echogenic mass of size 12.1*9.8*9.4 mm with no apparent vascularity on the left thorax (Figures 1, 2). The scan also revealed severe hydrops fetalis, cystic hygroma, generalised body oedema, mild hydrothorax, and ascites (Figures 3, 4). 

Chorionic villus sampling (CVS) was offered. Risks and benefits were explained, and the couple agreed to proceed with the invasive testing. Anti-D was given at the end of the procedure.

Uncomplicated CVS was performed on the same day as the scan. The foetal heartbeat was shown to the couple at the end of the procedure.

The initial result of quantitative fluorescent polymerase chain reaction (QF-PCR) aneuploidy for chromosomes 13, 18, 21, X, and Y was obtained four days post procedure, which was consistent with a single X chromosome and with normal complements for chromosomes 13, 18 and 21 and Y.

A comprehensive report was received a week later from the tertiary centre, which confirmed the results of QF-PCR, showing an abnormal female karyotype with monosomy X - Turner’s syndrome.

The findings were discussed with the couple, and they opted for medical termination of pregnancy. The patient had a termination of pregnancy for foetal anomaly (TOPFA) at 13+6 weeks and expelled the foetus completely. Anti-D was administered within 24 hours.

## Discussion

Foetal echogenic lungs are small bright spots in the foetal lungs diagnosed antenatally by ultrasound, indicating abnormal development. It is most commonly found in congenital cystic adenomatoid malformation (CCAM), pulmonary sequestration (PS), and congenital high airway obstruction syndrome (CHAOS). It occurs in 1 in 2500 pregnancies. The median age of diagnosis of CCAM, CHAOS, and PS was in the second trimester, between 16 and 17 weeks. In one literature review that included all cases with foetal echogenic lungs from the foetal medicine library over a period of 12 years, there were 193 cases with foetal echogenic lungs identified, which included nine cases of CHAOS, 170 cases of CCAM, and 14 cases of PS [2].

In CCAM, overgrowth of terminal bronchioles and lack of normal alveoli cause the formation of small cysts, which appear echogenic on ultrasound. CCAM can be classified into microcystic, macrocystic or mixed. It is the most common echogenic lung lesion in a foetus. It’s mostly unilateral, and it receives its blood supply from the pulmonary artery [3]. Once it's diagnosed, management is based on the type of CCAM, presence or absence of hydrops, and CCAM volume to head ratio. For microcystic lesions, treatment by percutaneous intratumoral laser coagulation or sclerotherapy has shown increased survival rates. For macrocystic lesions, thoraco-amniotic shunt is the first line of treatment. Maternal steroid therapy as a single course of 12 mg of betamethasone 24 hours apart or as multiple courses has significantly decreased the size of CCAM and increased the survival rate by 90% [4].

In PS, there is an extra lung tissue supplied by an aberrant branch of the aorta. In this condition, the colour Doppler can help visualise the blood supply from the aorta. Treatment involves ablation of this aberrant blood supply from the aorta via laser, ex utero intrapartum treatment (EXIT) procedure [5], and open foetal surgery.

CHAOS happens because of laryngeal stenosis, atresia, or tracheal atresia. When a major airway is blocked, fluid builds up distal to the blockage, and it can cause the appearance of increased echogenicity. The massively enlarged lungs cause cardiac and superior mediastinal compression, resulting in a secondary foetal hydrops and foetal/neonatal death. The EXIT procedure to perform a tracheostomy is the only postnatal treatment option to increase the survival rate [6].

Another study reported 48 cases of foetal echogenic lungs over a period of 10 years in a tertiary foetal medicine unit in the UK. 90% of the cases were CCAM, and 10% of them were PS [7]. In another study conducted in India, there were 40 cases of foetal echogenic lungs identified over a period of five years, which were discovered between 16- and 29-weeks’ gestation. Of the 40 cases, 70% were CCAM, and 15% were PS and CHAOS each [8]. CCAM and PS are benign compared to CHAOS. Many echogenic lesions resolve in utero, and the presence of co-existing abnormalities like hydrops will determine the outcome of such pregnancies. In the absence of hydrops, CCAM and PS have a good prognosis.

In another study, which studied the association between the bilateral foetal echogenic lung lesions detected during anomaly scans and chromosomal abnormalities, there were five cases detected over a period of two years, and four of them were positive for trisomy 21, and one had a normal karyotype [9]. The association of foetal echogenic lungs with Turner's syndrome, especially in the first trimester, is an unusual finding, and to date, there has been only one case reported, and the current case is the second. Turner’s syndrome was first presented by a German paediatrician, Otto Ullrich, in 1930, based on a girl exhibiting a clinical picture of Turner’s syndrome. Eight years later, the condition was explained by an Oklahoma physician; hence, this condition was later known as Ullrich-Turner syndrome. It's a sex chromosomal disorder affecting females in which there is an intact X chromosome along with a partially or completely absent second sex chromosome [10].

Incidence is 1 in 2000 to 1 in 2500 live female births [11]. The incidence may seem to be higher in the antenatal period, as the majority terminate such pregnancies or abort spontaneously. Though the advances in the diagnosis of Turner’s syndrome via ultrasound detection of syndromic features have increased, the true incidence is still unknown because many patients with mild phenotypes can go undiagnosed until adolescence. It’s not an inherited disorder; it occurs as a random event during the formation of reproductive cells in one parent. Prenatal diagnosis of Turner’s syndrome includes the presence of the following features in ultrasound: increased nuchal translucency, nuchal cystic hygroma, cardiac anomalies like coarctation of the aorta, brachycephaly, horseshoe kidney, polyhydramnios, oligohydramnios, or nonimmune foetal hydrops. In those who have the presence of two or more of the above-listed ultrasound features, genetic testing via chorionic villus sampling or amniocentesis should be offered. Noninvasive prenatal testing using cell-free foetal DNA and preimplantation genetic testing in women who are planning with their own oocytes is also advocated.

Diagnosis of Turner's syndrome is more likely with the presence of ultrasound features mentioned previously than with the presence of foetal echogenic lungs alone. The first case, which was reported, was diagnosed by the foetal medicine team at 14 weeks of gestation in a 28-year-old nulliparous woman. She was referred to FMU in view of her suspected cystic hygroma at her routine 12-week nuchal translucency scan. This baby also had similar ultrasound features to those in the present case report. The couple underwent termination of pregnancy following the confirmed diagnosis of Turner’s syndrome based on amniotic fluid karyotype at 15 weeks of gestation. In the report, the only variant was that the presence of echogenic lung lesions was noted bilaterally [1]. Thus, it is very crucial to have an early referral to a tertiary unit in case of such findings for further management.

## Conclusions

Foetal echogenic lungs detected antenatally are found to be associated with chromosomal abnormalities like Down’s syndrome and Turner’s syndrome. The prognosis is favourable in the absence of foetal hydrops. The insights provided in this report may assist the clinicians, genetic counsellors, and prenatal care team as they enlighten the importance of early detection and intervention. It is important for healthcare professionals to promptly refer to a tertiary foetal medicine unit when foetal echogenic lungs are identified during the first trimester for comprehensive evaluation and diagnosis, and it also provides the opportunity to plan appropriate management for the best possible outcomes for the foetus.
